# Monocyte Chemoattractant Protein 1 Promotes VEGF-A Expression in OSCC by Activating ILK and MEK1/2 Signaling and Downregulating miR-29c

**DOI:** 10.3389/fonc.2020.592415

**Published:** 2020-11-27

**Authors:** Ming-Yu Lien, An-Chen Chang, Hsiao-Chi Tsai, Ming-Hsui Tsai, Chun-Hung Hua, Shih-Ping Cheng, Shih-Wei Wang, Chih-Hsin Tang

**Affiliations:** ^1^School of Medicine, China Medical University, Taichung, Taiwan; ^2^Division of Hematology and Oncology, Department of Internal Medicine, China Medical University Hospital, Taichung, Taiwan; ^3^Translational Medicine Center, Shin-Kong Wu Ho-Su Memorial Hospital, Taipei, Taiwan; ^4^Department of Medical Research, MacKay Memorial Hospital, Taipei, Taiwan; ^5^Department of Otolaryngology, China Medical University Hospital, Taichung, Taiwan; ^6^Department of Medicine, Mackay Medical College, New Taipei, Taiwan; ^7^Department of Surgery, MacKay Memorial Hospital, Taipei, Taiwan; ^8^College of Pharmacy, Graduate Institute of Natural Products, Kaohsiung Medical University, Kaohsiung, Taiwan; ^9^Department of Biotechnology, College of Health Science, Asia University, Taichung, Taiwan; ^10^Chinese Medicine Research Center, China Medical University, Taichung, Taiwan

**Keywords:** monocyte chemoattractant protein-1, vascular endothelial growth factor A, miR-29c, oral squamous cell carcinoma, angiogenesis

## Abstract

Oral squamous cell carcinoma (OSCC) is an aggressive tumor that has a poor prognosis, with high levels of local invasion and lymph node metastasis. Vascular endothelial growth factor A (VEGF-A) plays essential roles in OSCC tumor angiogenesis and metastasis. Monocyte chemoattractant protein-1 (MCP-1, CCL2) is implicated in various inflammatory conditions and pathological processes, including oral cancer. The existing evidence has failed to confirm any correlation between MCP-1 or VEGF-A expression and OSCC angiogenesis. In this study, high expression levels of MCP-1 and VEGF-A were positively correlated with disease stage in patients with OSCC. In oral cancer cells, MCP-1 increased VEGF-A expression and subsequently promoted angiogenesis; miR-29c mimic reversed MCP-1 activity. We also found that MCP-1 modulated VEGF-A expression and angiogenesis through CCR2/ILK/MEK1/2 signaling. *Ex vivo* results of the chick embryo chorioallantoic membrane (CAM) assay revealed the angiogenic qualities of MCP-1, with increased numbers of visible blood vessel branches. Our data suggest that MCP-1 is a new molecular therapeutic target for the inhibition of angiogenesis and metastasis in OSCC.

## Introduction

Oral squamous cell carcinoma (OSCC) is an aggressive oral epithelial neoplasm that has a low overall 5-year survival rate of 50–60% (1), largely because of the development of metastasis (2). Research has therefore tended to concentrate on therapeutic strategies that interrupt the metastatic process in advanced-stage OSCC (3). OSCC metastasis is a complex process that is largely driven by angiogenesis (4, 5). Few therapeutic options are available for OSCC patients with metastatic disease (4). The production of monocyte chemotactic protein-1 (MCP-1, also known as CCL2) in the tumor microenvironment is recognized as having a crucial role in the growth, dissemination, and metastasis of head and neck cancer (6). The importance of targeting MCP-1 in metastatic disease has been highlighted in experimental models (7). MCP-1 can accelerate breast cancer metastasis by promoting angiogenesis in a vascular endothelial growth factor (VEGF)-A-dependent manner (8). MCP-1 is also involved in the migration and invasion of a variety of different types of cancer cells, such as breast, prostate, glioblastoma, ovarian, bladder, and chondrosarcoma (7). Some evidence suggests that MCP-1 might be involved in the development of OSCC (4, 9), including inducing changes in epithelial-mesenchymal transition (EMT) markers *via* paracrine or autocrine signaling (3), but the exact mechanism remains unclear.

OSCC is characterized by genomic and epigenomic alterations (10), although the mechanisms of OSCC tumorigenesis remain unclear. MicroRNAs (miRNAs) are small, non-coding RNAs capable of regulating gene expression by binding to the 3′-untranslated region (UTR) of target genes (11, 12) and are implicated in the regulation of tumor metastasis due to their influence upon cancer cell proliferation, migration, and angiogenesis (13). Dysregulated miRNA expression has been considered to play a role in OSCC progression and metastasis (14), but it remains to be explained as to how miRNAs mediate MCP-1–mediated VEGF-A expression in OSCC.

In this study, we demonstrate that MCP-1 promotes VEGF-A expression in OSCC by activating integrin-linked kinase (ILK) and MEK1/2 signaling and downregulating miR-29c expression, all of which subsequently enhances VEGF-A-induced tumor angiogenesis.

## Materials and Methods

### Materials

Human recombinant monocyte chemoattractant protein 1 (MCP-1) protein was purchased from PeproTech (Rocky Hill, NJ, USA). Anti-rabbit and anti-mouse IgG-conjugated horseradish peroxidase, rabbit polyclonal antibodies (specific for p-GSK3β (Santa Cruz sc-135653) and GSK3β (Santa Cruz sc-9166)), and mouse monoclonal antibodies (specific for VEGF-A (Santa Cruz sc-7269), β-actin (Santa sc-47778), MCP-1 (Santa Cruz sc-32771), ILK (Santa Cruz sc-20019), p-MEK1/2 (Santa Cruz sc-271914), MEK1/2 (Santa Cruz sc-6250), p-ERK1/2 (Santa Cruz sc-7383), and ERK1/2 (Santa Cruz sc-1647)) were purchased from Santa Cruz Biotechnology (Santa Cruz, CA, USA). The control miRNA and miR-29c mimic were purchased from Life Technologies (Carlsbad, CA, USA). Lipofectamine 2000 was purchased from Invitrogen (Carlsbad, CA, USA). All other chemicals were purchased from Sigma-Aldrich (St. Louis, MO, USA).

### Cell Culture

The human OSCC cell line used in this study was SCC4, and was purchased from the Bioresource Collection and Research Center (BCRC) (Hsinchu, Taiwan). Cells were cultured in 10% DMEM+F-12 medium and maintained at 37°C in a humidified atmosphere of 5% CO_2_. Human endothelial progenitor cells (EPCs) were provided by Dr. Wang Shih Wei. The EPCs were seeded onto 1% gelatin-coated plasticware and grown in endothelial cell growth medium MV2 with 20% FBS at 37°C in an atmosphere of 5% CO_2_.

### Immunohistochemical (IHC) Staining

Before proceeding with the staining protocol, the tissue array sections were deparaffinized and hydrated in xylene and graded ethanol solutions in distilled water. IHC analysis was performed according to the conditions of our previous protocol (15). Human MCP-1 or VEGF-A antibody was applied at 4°C overnight. The results were scored by accounting for the percentage of positive detection and intensity of the staining. Quantitative data were obtained using ImageJ software.

### Western Blot Analysis

After washing the cell culture dish with PBS, we added ice-cold RIPA lysis buffer containing protease inhibitors to lysate the cells. SDS-PAGE gels were used to separate protein samples, which were transferred to PVDF membranes according to our previously described methodology (15).

### ILK Kinase Activity Assay

ILK kinase activity was analyzed with Nonidet P-40 (NP-40) lysis buffer (16) and a GSK-3β phosphorylation assay was performed by Western blot analysis, as previously reported (17). Western blot visualized the phosphorylation of GSK-3β. Anti-ILK was used as a loading control.

### Quantitative Real-Time PCR

Quantitative real-time PCR was performed using the StepOnePlus™ sequence detection system, under the established protocol (18–20). VEGF-A primer sequences used were forward 5'-GCAGAATCATCACGAAGTGG-3' and reverse 5'-GCATGGTGATGTTGGACTCC-3'. The Mir-X™ miRNA First-Strand Synthesis and SYBR^®^ qRT-PCR kits detected miRNA expression. The specific primer sequence for miR-29c was 5'-TAGCACCATTTGAAATCGGTTA-3'. GAPDH or U6 snRNA expression level was used as the endogenous control for normalization purposes.

### ELISA

Cells were seeded in 6-well plates for 24 h and then the culture medium (CM) was changed to serum-free medium. Some cells were left untreated (controls), while others were treated only with MCP-1 for 24 h. Other cells were treated with pharmacological inhibitors or transfected with miRNA mimic for 30 min, then with MCP-1 for 24 h. The CM was then collected from all cells and VEGF-A concentrations were detected using the human VEGF-A/VEGF Quantikine ELISA kit (R&D Systems, MN, USA), according to the manufacturer’s protocol.

### Tube Formation Assay

The 48-well plates were coated with Matrigel (100 μl/well) before use. EPC cells (1×10^4^) were seeded into each well in 50% MV2 medium and 50% CM, then incubated for 16 h at 37°C. Quantitative data were obtained using ImageJ software.

### Migration Assay

The migration assay was performed in a 24-well Transwell cell culture chamber with 8.0-μm pore size inserts. EPCs (2×10^4^) were seeded onto the upper chamber and incubated in the lower chamber with 50% MV2 medium and 50% CM at 37°C in 5% CO_2_ for 24 h. Migratory ability was assayed using the procedure described in our previous publication (15).

### Luciferase Activity Assay

Cells were seeded onto 24-well plates and transiently transfected with wild-type- or mutant-type-VEGF-A 3'-UTR luciferase plasmids. Luciferase activity was assayed using our previously described procedure (21).

### Chick Chorioallantoic Membrane (CAM) Assay

The CAM assay detected the influence of MCP-1 on angiogenesis. We used 10-day-old fertilized chick embryos, with minor modifications from the procedures described in our previous study (15). The eggs were incubated in a humidified atmosphere of 80–90% at 38°C for 7 days. An approximate 1-cm opening was created in the air sac of each egg, into which 50 μl of CM (suspended in 50 μl Matrigel) was added, before the shells were covered with adhesive tape and the eggs were incubated for a further 72 h. Chorioallantoic membranes were collected for microscopy and photographic documentation. We manually counted the numbers of blood vessel branches within the defined area of the membrane, to form the angiogenic index.

### Statistical Analysis

Data are expressed as the mean ± SD. Between-group differences were analyzed using the Student’s *t*-test of variance and one-way analysis of variance (ANOVA) was used to compare means between two or more groups. The difference was considered significant if the *p* value was less than 0.05. Each experimental procedure was independently repeated three times, with similar results.

## Results

### Clinical Significance of MCP-1 Expression in Oral Squamous Cell Carcinoma

**Figure 1:**
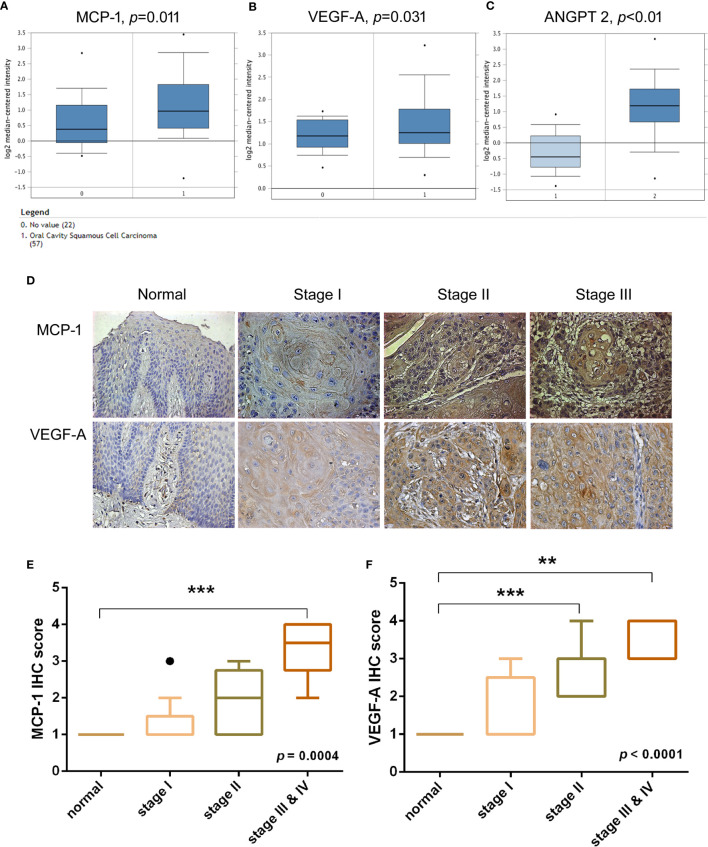
Clinical significance of MCP-1 in angiogenesis of oral squamous cell carcinoma. **(A–C)** OSCC tissue and adjacent normal tissue data from the Oncomine database (https://www.oncomine.org) were analyzed for MCP-1, VEGF-A, and ANGPT2 expression. **(D)** IHC photographs of tissue array sections (OR601) immunostained with anti-MCP-1 or anti-VEGF-A antibody. **(E, F)** IHC results were scored on a scale of 1–5 for staining intensity. Levels of MCP-1 and VEGF-A expression correlated with OSCC clinical grade. **p < 0.005 , ***p < 0.0005 compared with controls. **(A–C, E)** are box-and-whisker plots that plot outliers as individual points (dots).

Previous research has demonstrated that VEGF-A and angiopoietin 2 (ANGPT2) are critical players in tumor angiogenesis, while VEGF-A has been considered the primary factor driving the expansion of the tumor angiogenesis (22, 23). To confirm the clinical significance of MCP-1 in OSCC angiogenesis, we obtained data from the Oncomine database, which contains 65 gene chip datasets, 4700 chips, and 480 million gene expression data (24, 25). We found higher levels of MCP-1, VEGF-A, and ANGPT2 expression in tissue from OSCC patients compared with normal healthy controls (**Figures 1A–C**). Furthermore, IHC staining demonstrated that higher MCP-1 and VEGF-A expression correlated with higher clinical disease stage (**Figures 1D–F**). These findings suggest that MCP-1 has an important role in OSCC progression.

### MCP-1 Promotes Angiogenesis in Human OSCC Cells *via* VEGF-A-Dependent Signaling

**Figure 2:**
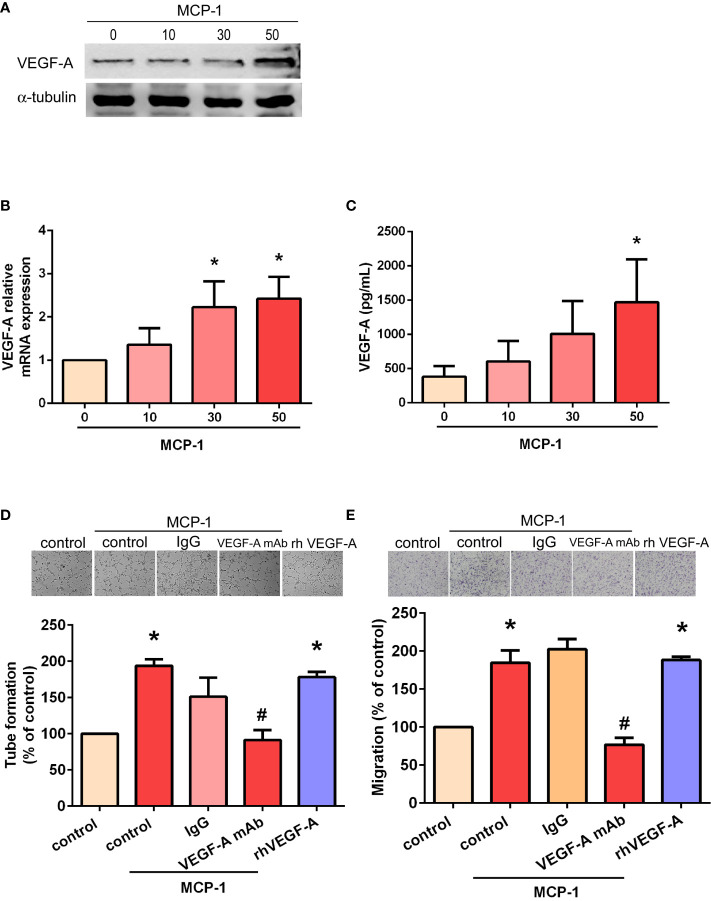
MCP-1 promotes human OSCC cell angiogenesis through the VEGF-A-dependent pathway. Cells were incubated with MCP-1 (0–50 ng/ml) for 24 h and VEGF-A expression was determined by Western blot **(A)**, RT-qPCR **(B)**, and ELISA assays **(C)**. The CM was collected and used to treat the EPCs for 24 h. We used the tube formation assay to examine capillary-like structure formation **(D)** and the Transwell assay to examine cell migration **(E)**. *p < 0.05 compared with controls; ^#^*p* < 0.05 compared with MCP-1–treated controls. Each experimental procedure was independently repeated three times, with similar results.

We sought to determine whether MCP-1–induced angiogenesis of OSCC cells involves VEGF-A signaling. As shown in **Figures 2A-C**, incubation of SCC4 cells with human recombinant MCP-1 significantly increased VEGF-A mRNA expression and protein secretion. We found that incubation of EPCs with CM from MCP-1–treated OSCC cells promoted EPC tube formation and migration; these activities were abolished when 5 μg/ml of VEGF-A monoclonal antibody (mAb) was added (**Figures 2D, E**). These results confirmed that MCP-1 promotes angiogenesis through the VEGF-A-dependent pathway.

### MCP-1 Promotes VEGF-A Expression and Angiogenesis *via* the CCR2 Receptor

**Figure 3:**
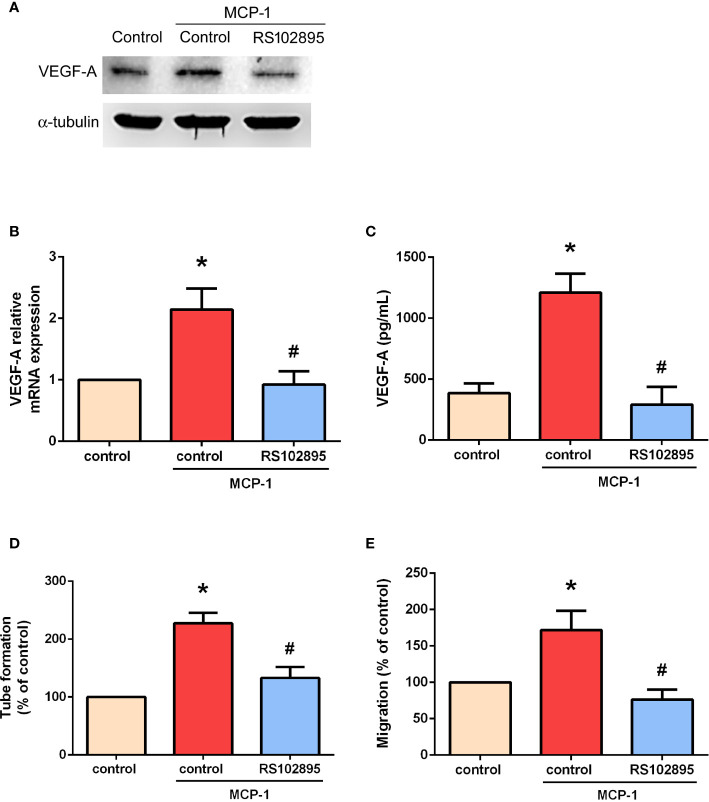
MCP-1 promotes VEGF-A expression and angiogenesis *via* the CCR2 receptor. Cells were treated with a CCR2 antagonist (RS102895; 100 ng/ml) for 30 min and then stimulated with MCP-1 (50 ng/ml) for 24 h. VEGF-A expression was measured by Western blot **(A)**, RT-qPCR **(B)**, and ELISA assays **(C)**. Cells were treated with RS102895 (100 ng/ml) for 30 min and then stimulated with MCP-1 (50 ng/ml) for 24 h, then CM was collected. After treating the EPCs with CM for 24 h, capillary-like structure formation was examined using the tube formation assay **(D)** and cell migration was examined with the Transwell assay **(E)**. *p < 0.05 compared with controls; ^#^*p* < 0.05 compared with MCP-1–treated controls. Each experimental procedure was independently repeated three times, with similar results.

Targeting MCP-1/CCR2 signaling may alter the tumor microenvironment and enhance angiogenesis in cancer (26, 27). We therefore used RS102895, a potent CCR2 antagonist, to evaluate whether MCP-1–induced stimulation of VEGF-A expression and angiogenesis is influenced by CCR2. RS102895 treatment significantly reduced protein and mRNA expression levels (**Figures 3A–C**). RS102895 also significantly inhibited EPC tube formation and migration (**Figures 3D, E**). The CCR2 receptor therefore plays an important role in MCP-1–induced stimulation of VEGF-A expression and angiogenesis.

### The ILK/MEK1/2 Signaling Pathway Is Involved in MCP-1–Induced VEGF-A Expression and Angiogenesis

**Figure 4:**
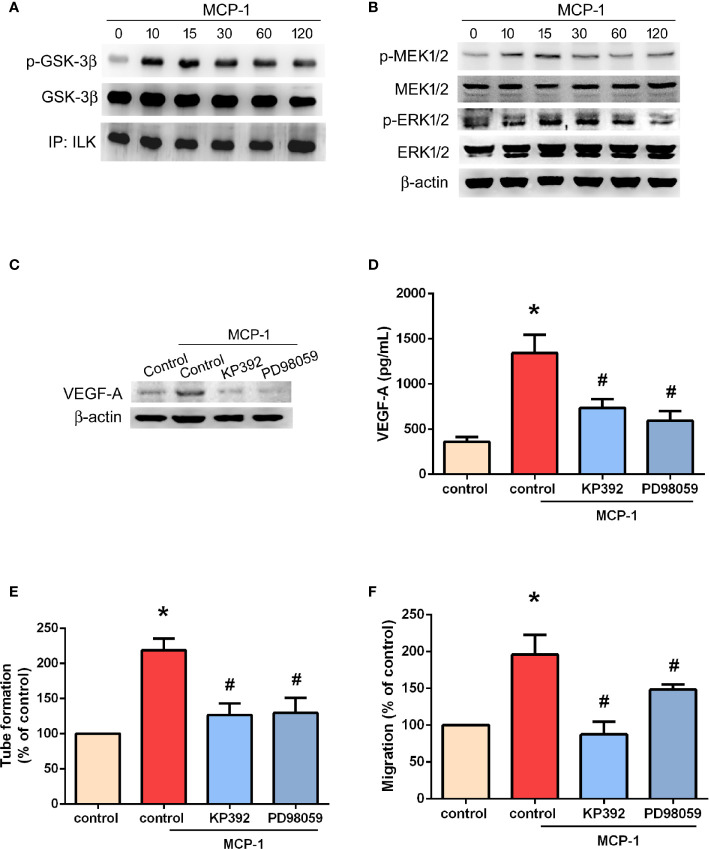
ILK/MEK1/2 signaling is involved in MCP-1–induced VEGF-A expression and angiogenesis. Cells were incubated with MCP-1 (50 ng/ml) for the indicated time intervals; **(A)** Cell lysates were immunoprecipitated (IP) with ILK antibodies prior to Western blotting using p-GSK-3β, GSK-3β, and ILK antibodies. **(B)** MEK1/2, and ERK1/2 activation was examined by the Western blot assay. **(C, D)** Cells were treated with an ILK inhibitor (KP392; 10 μM) or MEK1/2 inhibitor (PD98059; 10 μM) for 30 min, then stimulated with MCP-1 (50 ng/ml) for 24 h. VEGF-A expression was measured by Western blot and ELISA assays. **(E, F)** Cells were treated with a KP392 (10 μM) or PD98059 (10 μM) for 30 min, then stimulated with MCP-1 (50 ng/ml) for 24 h. CM was applied to EPCs for 24 h, then capillary-like structure formation was examined by the tube formation assay **(E)** and cell migration by the Transwell assay **(F)**. *p < 0.05 compared with controls; ^#^*p* < 0.05 compared with MCP-1–treated controls. Each experimental procedure was independently repeated three times, with similar results.

ILK is considered to be crucial for tumor angiogenesis (28). The GSK-3 crosstide peptide is a substrate of ILK, and phosphorylation of GSK-3β is regulated by ILK in many cells (29). Here, we found that MCP-1 promotes ILK kinase activity by promoting the phosphorylation of GSK-3β (**Figure 4A**) and mitogen-activated protein kinase kinase (MEK 1/2) in a time-dependent manner (**Figure 4B**). In addition, the phosphorylation of extracellular signal-regulated protein kinase (ERK1/2) was also promoted in a time-dependent manner (**Figure 4B**). Pretreatment with KP-392 (a potent and selective inhibitor of ILK) or PD98059 (a potent and selective inhibitor of MEK1/2) significantly reduced MCP-1–induced increases in VEGF-A expression (**Figures 4C, D**), as well as EPC tube formation and migration (**Figures 4E, F**). These results indicate that MCP-1 promotes VEGF-A expression and angiogenesis in OSCC cells through ILK and MEK1/2 signaling.

### MiR-29c Is a Key miRNA in MCP-1–Promoted VEGF-A Expression and Angiogenesis

**Figure 5:**
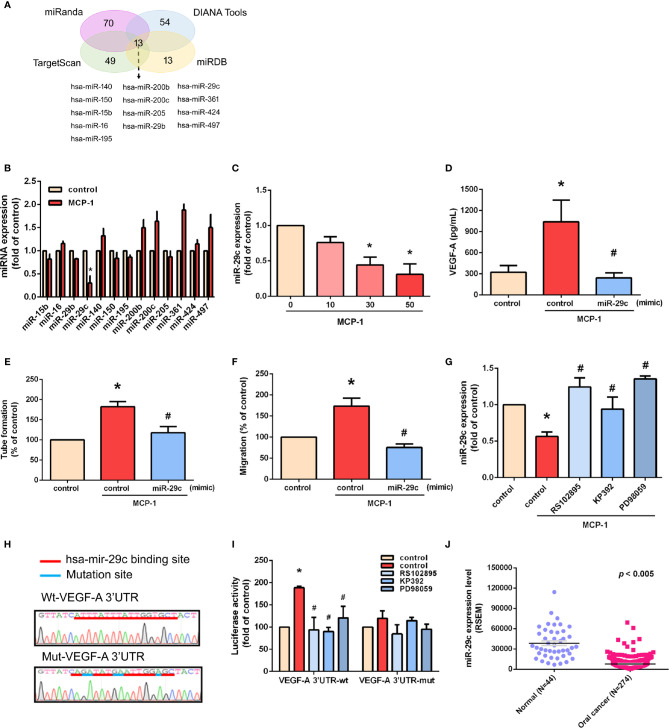
miR-29c is a key miRNA in MCP-1–induced stimulation of VEGF-A expression and angiogenesis. **(A)** MiRNA target prediction program software was used to identify miRNAs that potentially bind to the VEGF-A 3'-UTR. **(B)** Cells were incubated with MCP-1 for 24 h and expression levels of the indicated miRNAs were subjected to qPCR analysis. **(C)** Cells were incubated with MCP-1 (0–50 ng/ml) for 24 h and miR-29c expression was determined by qPCR. **(D)** Cells were transfected with miR-29c mimic (10 nM) then incubated with MCP-1 for 24 h. VEGF-A expression was measured by ELISA. **(E, F)** CM was applied to EPCs for 24 h then capillary-like structure formation was examined by the tube formation assay and cell migration by the Transwell assay. **(G)** Cells were treated for 30 min with RS102895 (100 ng/ml), KP392 (10 μM), or PD98059 (10 μM) then incubated with MCP-1 for 24 h. miR-29c expression was examined by qPCR analysis. **(H)** The wild-type or mutant VEGF-A 3'-UTRs containing the miR-29c binding site was inserted into the pmirGLO vector. **(I)** Cells were transfected with wt-VEGF-A-3'-UTR or mut-VEGF-A-3'-UTR plasmid for 24 h, then stimulated for 30 min with RS102895 (100 ng/ml), KP392 (10 μM), or PD98059 (10 μM), followed by MCP-1 treatment for 24 h; luciferase activities were measured. **(J)** TCGA database records were analyzed for levels of miR-29c expression in tumor tissue and adjacent normal tissue. *p < 0.05 compared with controls; ^#^*p* < 0.05 compared with MCP-1–treated controls. Each experimental procedure was independently repeated three times, with similar results.

We used open-source miRNA prediction software (miRanda, DIANA Tools, TargetScan, and miRDB) to identify 13 candidate miRNAs that target VEGF-A mRNA (**Figure 5A**). Of all miRNAs, miR-29c was the most downregulated after MCP-1 treatment (**Figure 5B**). Exogenous MCP-1 dose-dependently and significantly inhibited miR-29c expression (**Figure 5C**). Transfection of cells with miR-29c mimic reduced the ability of MCP-1 to enhance VEGF-A expression (**Figure 5D**), EPC tube formation (**Figure 5E**) and EPC migration (**Figure 5F**). CCR2/ILK/MEK1/2 signaling was involved in the effects of MCP-1 on miR-29c expression (**Figure 5G**). To confirm that miR-29c directly binds to the 3'-UTR of VEGF-A and inhibits VEGF-A mRNA translation, we constructed wild- and mutant-type luciferase reporter vectors harboring VEGF-A 3'-UTR (**Figure 5H**), then transfected these vectors into OSCC cells. We found that MCP-1 increased luciferase activity in the wild-type plasmid *via* CCR2, ILK, and MEK1/2 signaling, but no such effect was observed with the mutant-type plasmid (**Figure 5I**). When we examined tumor samples from the TCGA database, we found much lower levels of miR-29c expression in oral cancer tissue than in adjacent normal tissue (**Figure 5J**). These data indicate that miR-29c suppresses VEGF-A protein expression by binding to the 3′-UTR of the human *VEGF-A* gene *via* CCR2/ILK/MEK1/2 signaling.

### Effects of MCP-1 on VEGF-A-Induced Angiogenesis in the CAM Model

**Figure 6:**
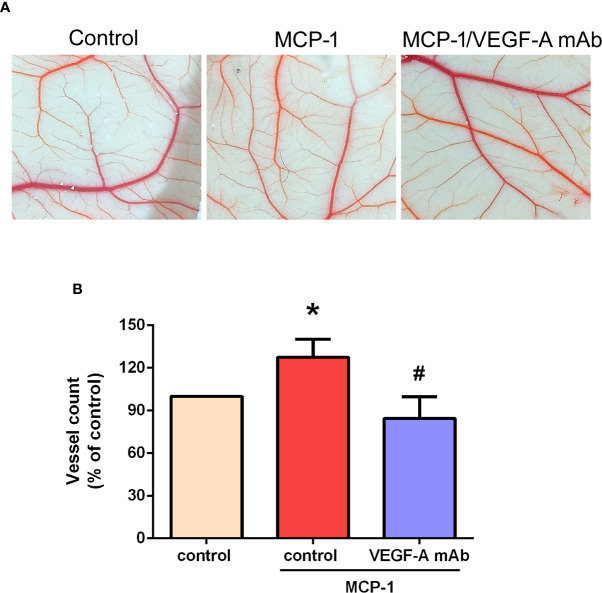
Effects of MCP-1 on VEGF-A-induced angiogenesis in the CAM model. **(A)** Cells were incubated with or without MCP-1 (50 ng/ml) for 24 h, or treated for 30 min with VEGF-A antibody (5 μg/ml) followed by MCP-1 (50 ng/ml) for 24 h. CM was collected and suspended in Matrigel, then incubated with chick embryos (n=5 in each group) for another 72 h. Chorioallantoic membranes were collected for microscopy and photographic documentation. **(B)** Angiogenesis was quantified by manually counting the number of blood vessel branches. *p < 0.05 compared with controls; ^#^*p* < 0.05 compared with MCP-1–treated controls. Each experimental procedure was independently repeated three times, with similar results.

We used the CAM assay to characterize the effect of MCP-1 on VEGF-A-induced angiogenesis. As illustrated in **Figures 6A, B**, we found that the CM collected from MCP-1–treated OSCC cells enhanced the number of blood vessel branches compared with untreated cells, whereas MCP-1–enhanced blood vessel growth was significantly reduced in cells pretreated with VEGF-A mAb. These results indicate that MCP-1 promotes angiogenesis *ex vivo*.

## Discussion

Angiogenesis is a critical feature of OSCC progression (30). Some evidence suggests the involvement of MCP-1 in the development of OSCC (4, 9), including the induction of epithelial-mesenchymal transition (3), although the exact mechanisms remain unclear. Our study evidence indicates that MCP-1 is an important player in OSCC angiogenesis, as we observed a correlation between higher levels of MCP-1 expression and OSCC disease status.

Dysregulation of chemokine or chemokine receptors has been linked to many diseases, especially those associated with cancer (31). In various human tissues, MCP-1 preferentially binds to the CCR2 receptor and has been implicated in the promotion of metastasis (27). Here, we found that inhibiting CCR2 functioning dramatically suppressed VEGF-A-induced angiogenesis. The ILK kinase is considered to be an important regulator in many key biological processes and is known to enhance OSCC tumorigenesis (32, 33). Knockdown of ILK reportedly inhibits OSCC proliferation, invasion and metastasis of xenograft tumors *in vivo* (34). In this study, we identified a new function of ILK in OSCC, namely, its regulation of MCP-1–mediated VEGF-A-associated angiogenesis. MEK1/2 has been shown to regulate the development and progression of cancer-associated angiogenesis (35, 36). The function of MEK1/2 in OSCC mostly involves cancer proliferation, chemoresistance, and invasion/migration (37–39). Here, we report that MEK1/2 inhibitors antagonized MCP-1–induced VEGF-A expression. Incubation of OSCC cells with MCP-1 promoted, ILK and MEK1/2 phosphorylation, suggesting that their activation plays a crucial role in MCP-1–stimulated VEGF-A production and angiogenesis in OSCC cells.

Evidence of significant downregulation of miR-29c in many cancers suggests that miR-29c acts as a suppressor miRNA in these diseases (40, 41). In laryngeal squamous cell carcinoma, significant correlations have been observed between decreased miR-29c expression and the smoking index, tumor size, tumor site, tumor differentiation, T classification, TNM stage of laryngeal squamous cell carcinoma, and lymph node metastasis (42). Interestingly, miR-29c appears to contribute to a paracrine cross-talk mechanism between cancer-secreted insulin-like growth factor 2 (IGF2) and VEGF expression in cancer-associated fibroblasts (CAFs), with evidence indicating that higher IGF2 expression in cancer cells combined with increased VEGF expression in CAFs is associated with an unfavorable prognosis in esophageal squamous cell carcinoma (43). In analyses of predictive algorithms, miR-29c has a high predictive score for *VEGF* 3′-UTR-binding sites and is one of the most significantly downregulated miRNAs in IGF2-treated CAFs (43). Moreover, miR-29c overexpression has been linked to a significant and consistent downregulation of VEGF in CAFs (43). It is well known that miR-29c acts as an anti-angiogenic agent by potently inhibiting VEGF-A (44). The expression of hsa-miR-29c is related to its DNA copy number and is differentially expressed in metastatic and nonmetastatic samples taken from patients with OSCC (45). In this study, we found that among all identified miRNAs, the level of miR-29c expression was reduced by the greatest extent after MCP-1 stimulation. Co-transfection with miR-29c mimic significantly reduced MCP-1–induced VEGF-A expression, EPC tube formation and migration. We also found that miR-29c directly represses VEGF-A protein expression by using ILK/MEK1/2 signaling to bind to the 3'-UTR of the human *VEGF-A* gene, and negatively regulating VEGF-A-mediated angiogenesis. Importantly, levels of miR-29c expression may serve as a biomarker of carcinogenesis in OSCC, as miR-29c has shown good discriminatory power for differentiating between OSCC and cancer-free tissue, as well as between tumor-adjacent and noncancerous tissue (46). Notably, the lack of a significant difference between miR-29c expression in tumor and tumor-adjacent tissues suggests a field cancerization effect in OSCC, whereby the dysregulation of miR-29c expression in tumor-adjacent tissue may promote oral carcinogenesis in that environment (46). In addition, miR-29c targets the phosphate and tensin homolog (*PTEN*) and *DICER1* genes, both of which contribute to oral carcinogenesis (46). In this respect, molecular analysis of miR-29c expression could be very important for OSCC prognosis. We suggest that combining miR-29c with other therapies that effectively target VEGF-A may improve the treatment of OSCC.

## Conclusions

**Figure 7:**
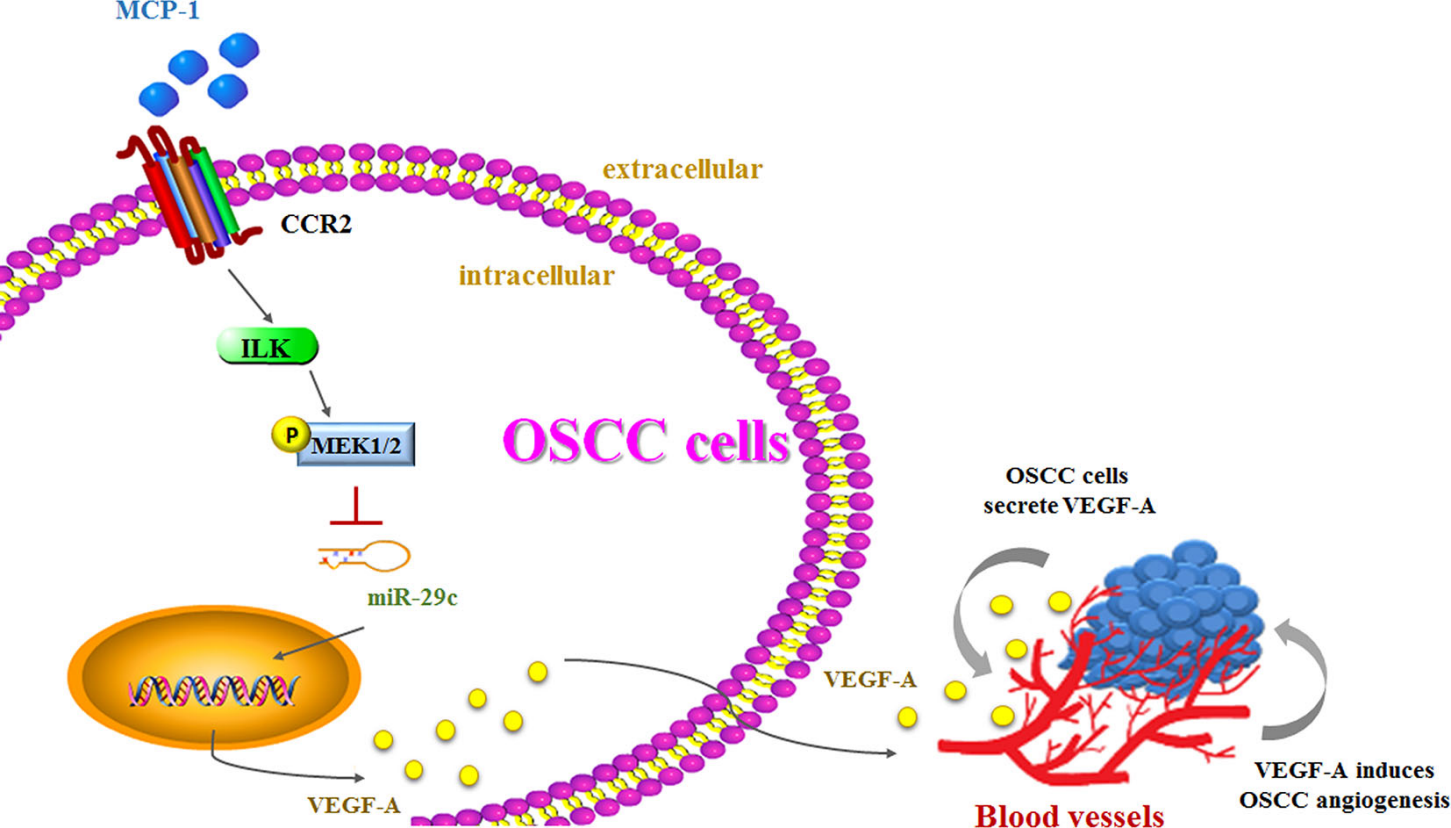
The schema depicts the involvement of signaling pathways in MCP-1–induced stimulation of VEGF-A expression and angiogenesis.

Higher MCP-1 expression levels in patients with OSCC are associated with more advanced disease. In cellular experiments, we found that MCP-1 promotes VEGF-A expression and angiogenesis by downregulating miR-29c expression *via* the ILK/MEK1/2 signaling pathways (**Figure 7**). Our evidence indicates that MCP-1 may be a new molecular therapeutic target for inhibition of angiogenesis and metastasis in OSCC.

## Data Availability Statement

The raw data supporting the conclusions of this article will be made available by the authors, without undue reservation.

## Author Contributions

M-YL and A-CC designed the experiments and performed data analysis. M-YL, A-CC, and H-CT performed cell culture and *ex vivo* experiments. A-CC and H-CT performed data analysis. M-HT, C-HH, S-PC, and S-WW contributed to and discussed the research strategy and data interpretation. The paper was written by H-CT and edited by C-HT. All authors contributed to the article and approved the submitted version.

## Funding

This work was supported by grants from the Ministry of Science and Technology of Taiwan (MOST 108-2320-B-039-055; MOST 107-2320-B-039-019-MY3), China Medical University Hospital (DMR-109-205; CMU109-MF-44), and Shin Kong Wu Ho-Su Memorial Hospital (2020SKHBND001).

## Conflict of Interest

The authors declare that the research was conducted in the absence of any commercial or financial relationships that could be construed as a potential conflict of interest.
